# A systematic review on integration mechanisms in human and animal health surveillance systems with a view to addressing global health security threats

**DOI:** 10.1186/s42522-020-00017-4

**Published:** 2020-06-08

**Authors:** Janeth George, Barbara Häsler, Irene Mremi, Calvin Sindato, Leonard Mboera, Mark Rweyemamu, James Mlangwa

**Affiliations:** 1 Department of Veterinary Medicine and Public Health, Sokoine University of Agriculture, P.O. Box 3021, Morogoro, Tanzania; 2 SACIDS Foundation for One Health, Sokoine University of Agriculture, P.O. Box 3297, Morogoro, Tanzania; 3 Department of Pathobiology and Population Sciences, Veterinary Epidemiology, Economics, and Public Health Group, Royal Veterinary College, Hawkshead Lane, North Mymms, Hatfield, Hertfordshire, AL97TA UK; 4 National Institute for Medical Research, Tabora Research Centre, Tabora, Tanzania

**Keywords:** Health, Surveillance, Integration, Mechanism, Animal, Human, One health, Disease

## Abstract

**Background:**

Health surveillance is an important element of disease prevention, control, and management. During the past two decades, there have been several initiatives to integrate health surveillance systems using various mechanisms ranging from the integration of data sources to changing organizational structures and responses. The need for integration is caused by an increasing demand for joint data collection, use and preparedness for emerging infectious diseases.

**Objective:**

To review the integration mechanisms in human and animal health surveillance systems and identify their contributions in strengthening surveillance systems attributes.

**Method:**

The review followed the Preferred Reporting Items for Systematic Reviews and Meta-Analysis Protocols (PRISMA-P) 2015 checklist. Peer-reviewed articles were searched from PubMed, HINARI, Web of Science, Science Direct and advanced Google search engines. The review included articles published in English from 1900 to 2018. The study selection considered all articles that used quantitative, qualitative or mixed research methods. Eligible articles were assessed independently for quality by two authors using the QualSyst Tool and relevant information including year of publication, field, continent, addressed attributes and integration mechanism were extracted.

**Results:**

A total of 102 publications were identified and categorized into four pre-set integration mechanisms: interoperability (35), convergent integration (27), semantic consistency (21) and interconnectivity (19). Most integration mechanisms focused on sensitivity (44.1%), timeliness (41.2%), data quality (23.5%) and acceptability (17.6%) of the surveillance systems. Generally, the majority of the surveillance system integrations were centered on addressing infectious diseases and all hazards. The sensitivity of the integrated systems reported in these studies ranged from 63.9 to 100% (median = 79.6%, *n* = 16) and the rate of data quality improvement ranged from 73 to 95.4% (median = 87%, *n* = 4). The integrated systems were also shown improve timeliness where the recorded changes were reported to be ranging from 10 to 91% (median = 67.3%, *n* = 8).

**Conclusion:**

Interoperability and semantic consistency are the common integration mechanisms in human and animal health surveillance systems. Surveillance system integration is a relatively new concept but has already been shown to enhance surveillance performance. More studies are needed to gain information on further surveillance attributes.

## Background

Health surveillance is the systematic, continuous collection, collation, analysis, interpretation, and dissemination of epidemiological, economic and risk factor data from defined human or animal populations to inform decision making [1–3]. Surveillance helps decision-makers to manage disease prevention and control more effectively by providing timely and useful evidence for targeted action [4]. In animal health, surveillance serves four main objectives, namely demonstration of disease freedom, early detection of disease, case finding and measuring the level of disease [5]. Surveillance is categorized into active surveillance, passive surveillance and sentinel surveillance [6]. Types of surveillance may also be categorized to include early warning surveillance, indicator-based surveillance, hazard-specific surveillance, general surveillance, syndromic surveillance, event-based surveillance, risk-based surveillance, enhanced passive surveillance and participatory surveillance [2]. Surveillance systems have surpassed the initial emphasis on infectious diseases to include monitoring and forecast of a broad range of health determinants, such as risk behaviors, health care services, socioeconomic factors, outcomes of intervention programs, non-communicable diseases and environmental health [7].

According to the World Health Organization [8], an effective surveillance system must be able to perform the following functions: detection and notification of health events, collection and consolidation of pertinent data, investigation and confirmation of cases or outbreaks, routine analysis and creation of reports, feedback of information to those providing the data, feed-forward and reporting data to higher administrative levels. The World Organization for animal health (OIE) regards animal health surveillance as a tool to monitor disease trends, facilitate control of infection or infestation, and provide data for risk analysis in animal or public health in order to substantiate sanitary measures and to provide assurance to trading partners [3]. Animal health surveillance is recognized as a key element in predicting public health risks related to emerging zoonotic disease [9]. Environmental health surveillance is also an important component in predicting future outbreaks through monitoring environmental risk factors [10], yet it is often overlooked [11].

Various strategies can be used to solicit surveillance data such as periodic population-based surveys, sentinel surveillance, laboratory-based surveillance and integration of two or more surveillance programs or systems. Often, health surveillance systems rely upon data from varied sources with time lags between observed symptoms of the diseases, laboratory submission, results and communication to the appropriate authorities [12] thereby causing inefficiencies in the system and sub-optimal performance. To address this problem and to improve detection, reporting and response capabilities, surveillance system integrations have been promoted [9–12]. This is because health surveillance and preparedness for disease control and management require coordination and collaboration among various programs and wider range of expertise including front-line health care providers (veterinarians or clinicians), epidemiologists, information system specialists and laboratory personnel [13].

The term integration has been widely used in various fields including health, business management, engineering, transportation, and information technology. In these fields, integration is aimed at accelerating decision-making processes and improving coordination thereby increasing the efficiency of the system. However, depending on the context the term is used, there are different interpretations and outcome measurements. So far, there is no standard definition of system integration. For instance, in the engineering sciences, system integration involves the combination of hardware, software, products, services, processes, and humans [14]. In technology, system integration involves a complete system of business processes, managerial practices, organizational interactions, structural alignments and knowledge management [15]. The WHO defined integration in health service delivery as “the organization and management of health services so that people get the care they need, when they need it, in ways that are user-friendly, achieve the desired results and provide value for money” [16].

In health surveillance, system integration has been defined as the sum of all surveillance activities which add up to the broader surveillance system; it includes many functions using similar structures, processes, and personnel [17]. While integration within one sector is a common mechanism, “One Health” surveillance integration places emphasis on surveillance activities that span multiple sectors including human, animal and environmental health and benefit from cross-fertilization and exchange to promote health for all. One Health is defined as an a collaborative, multidisciplinary, and multi-sectoral approach that can address urgent, ongoing, or potential health threats at the human-animal-environment interface at subnational, national, global, and regional levels [18, 19]. Integration in health surveillance systems may include merging of health records database with surveillance system, sharing of databases with heterogeneous data to form common indicators or merging of surveillance activities and processes. During the past two decades, there have been a number of initiatives to integrate health surveillance systems using various mechanisms ranging from the integration of data sources to changing organizational structures and responses [10, 13].

Myerson categorized integration into four mechanisms, namely interconnectivity, interoperability, semantic consistency and convergent integration [15]. Interconnectivity includes the sharing of external devices or simply transferring files while the basic applications, functionality and uses all remain fairly specific with respect to their technologies and users with little or no integration at the function levels. In health surveillance, this kind of integration may be through the exchange of information between two systems in order to alert the authorities of any unusual disease event for appropriate action [20]. Interoperability is the ability of the system or its component to work with another while exploring the capabilities of both without special effort from the users [21]. For instance, animal health surveillance systems may interoperate with hospital medical records. It allows the systems to communicate, exchange data based on the standards and use information that has been exchanged [22]. Semantic consistency is directed towards the implementation of database management systems and sophisticated management reporting systems such as HealthMap and FAO EMPRES-i. The emphasis is on providing access to data and minimizing the potential for errors in human interpretation through the creation of standard data definitions and formats. Convergent integration involves the merging of technology with business processes, knowledge, and human performance. It is the highest and most sophisticated form of the integration state. Its key components include technology and data repository integrations, communication networks, embedding knowledge and human performance with the new processes and enabling technologies. Some of the examples of such integrations are evident in One Health and Integrated Disease surveillance and response strategies.

Often, the motivation behind systems integration revolves around technology, the need to produce better information for disease management and cost reduction, but the integration process may turn out to be inflexible and expensive to maintain [15]. Integration may not be a cure for inadequate resources [16]. In health surveillance, integration is driven by increasing demand for joint data collection and use [23] and preparedness for emerging infectious diseases [24, 25]. The International Health Regulations (IHR) 2005 require timely detection and response to outbreaks and suggested a combination of surveillance methods in addressing public health threats [21, 22]. Despite the need for systems integration in health surveillance, the overarching questions are still how much integration is optimal in terms of cost and effectiveness in addressing health challenges, what can be integrated, how to integrate and what factors to consider when integrating the system. Understanding the process of integration and assessing its impact requires systematic evaluation using empirical data. However, there are very few such studies and decisions regarding integration need to be made [26]. The objective of this paper was therefore, to identify and categorize mechanisms in which existing human and animal health surveillance systems have been integrated, assess the contribution of integrated systems in strengthening relevant surveillance attributes, and key aspects to consider in integration in order to address global health security threats.

## Methods

This review was guided by the following questions: (a) What are the existing integration mechanisms in animal and human health surveillance systems?; (b) To what extent have the integrations strengthened health surveillance systems attributes and added value to disease control strategies?; and (c) What are the important issues to consider in health surveillance systems integration? The authors acknowledge that One Health approach encompasses human, animal, environment and plants as previously defined [18, 19]. However, the review focused exclusively on the animal and human health surveillance systems. One health was regarded as one of the integration approach and that was the basis for the search strategy.

The review followed the Preferred Reporting Items for Systematic Reviews and Meta-Analysis Protocols (PRISMA-P) 2015 checklist [27]. The process included identification of search terms and searching of literature in relevant databases, application of inclusion and exclusion criteria, appraising of each study, data extraction using MS-Excel spreadsheet form (Excel 2010, Microsoft Corp., and Redmond, WA, USA) data synthesis and summarizing of information. The following search terms and Boolean operators were used: (Surveillance OR monitor*) AND (“animal health” OR “human health” OR “public health” OR “One Health”) AND (integrate*) AND (system). The databases searched were PubMed, HINARI, Web of Science, Science Direct and advanced Google search. The choice of databases considered the research questions and the percentage of the relevant documents from the search results. Respective index terms/search queries were used in order to generate relevant studies. Reference lists of primary articles were further searched for additional studies. The search included all studies in the English published between 1900 and 2018 and used quantitative, qualitative or mixed research methods.

Following the exclusion of duplicates, all articles found were screened. The screening was a two-stage process; the first stage was Title/abstract screening and the second stage was full paper screening. The following inclusion criteria were used: studies had to involve human health surveillance, animal health surveillance, or One Health surveillance systems and interventions and focus on integrated surveillance systems, describe integration designs of the system, or present the effects of the surveillance integration on surveillance systems attributes. The studies with abstracts without full text, not in English or newsletter articles were excluded. In order to ascertain the quality of included studies, the risk of bias was assessed using QualSyst Tool for qualitative and quantitative data [28].

Eligible articles were read and appraised independently by two authors (JG and IM) using the set criteria and relevant information was extracted using prepared data extraction sheets. In case of any disagreement, the consensus was reached through discussion. Data were extracted on two primary outcomes: i) Pre-defined integration mechanisms which were applied in the reviewed articles, and ii) surveillance system attributes. In order for the integrated surveillance system to be categorized, the assessment was done against the pre-defined mechanisms which are interconnectivity, interoperability, semantic consistency and convergent integration. A category was assigned to the particular article based on the description of the system as per pre-defined integration mechanisms above. The surveillance system attributes used were; simplicity, flexibility, data quality, acceptability, sensitivity, positive predictive value, representativeness, timeliness, cost-effectiveness, and stability (Table 1) [29]. The next step was to describe the added value of the systems integration in addressing target hazards and strengthening of surveillance system attributes. The target hazards included in the analysis were infectious diseases, non-communicable diseases, Antimicrobial resistance, injuries and other environmental risks and all-hazards (the combination of all mentioned hazards). From the eligible studies, important issues to consider in surveillance system integrations were extracted and presented through narrative synthesis.

**Table 1:** Surveillance attributes considered in the analysis

Attribute	Definition
Acceptability	The willingness of persons and organizations to participate in the surveillance system.
Cost-effectiveness	Relationship between the expected outcomes (such as the number of lives saved) and the costs of surveillance required to achieve this. May be expressed as a measure of efficiency, whereby the system operates at the least possible cost or makes the best use of available resources.
Data quality	Completeness and validity of the data recorded.
Flexibility	Ability to adapt to changing information needs or operating conditions with little additional time, personnel or allocated funds. Flexible systems can accommodate new health-related events, changes in case definitions or technology, and variations in funding or reporting sources.
Positive predictive value	The proportion of reported cases that actually have the infection/condition of interest.
Representativeness	The extent to which features of the population of interest (e.g. herd size, age, location) are reflected in the surveillance data that are collected.
Sensitivity	For endemic diseases, sensitivity refers to the proportion of cases of a disease detected by the surveillance system (this usually requires a gold standard test to indicate the actual number of cases). For non-endemic diseases, sensitivity refers to the ability of a surveillance system to detect disease outbreaks.
Simplicity	Refers to the surveillance system structure, ease of operation and flow of data through the system.
Stability	Reliability (function without failure) and availability (operational when needed)
Timeliness	Speed between steps in a surveillance system. For outbreak detection, timeliness refers to the time between exposure to the infectious agent and the initiation of interventions to control infection.

Source: Adopted from Drewe et al., 2012 [29]

## Results

### Overview of the search results

**Fig. 1:**
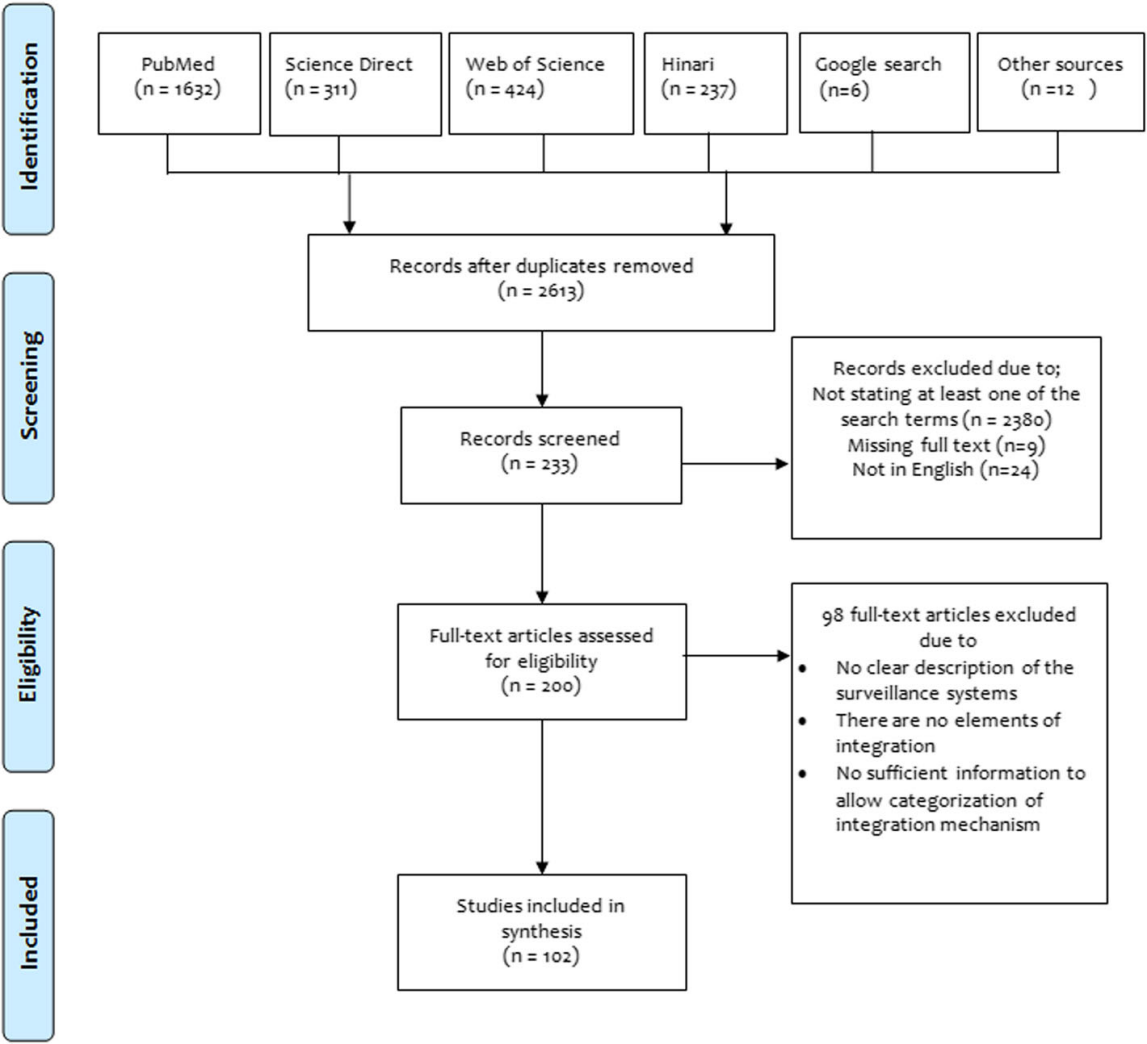
PRISMA flow diagram demonstrating articles selection process

A total of 2622 articles were found in the initial search, of which 9 duplicates were excluded. From the remaining 2613 articles, 2380 were excluded through initial title and abstract screening for not being relevant. Two hundred thirty three articles that went through the second screening process where 33 were excluded (as 24 were not in English language and 9 were not presented in full-texts). Two hundred full-text articles were assessed for eligibility whereby 98 were removed based on the specified inclusion and exclusion criteria. (Fig. 1). Finally, 102 articles met inclusion criteria and were therefore included in the synthesis (Table 2). The included studies were of average quality (Supplementary 1 and 2). For Qualitative studies, QualSyst score was 8–18 (mean = 12, *n* = 66) while for quantitative studies, the score was 15–20 (mean = 19, *n* = 36).

**Table 2:** Surveillance attributes and integration mechanisms extracted from the articles included in the review (*n* = 102 studies)^a^

Attribute	Mechanism	No. of articles	References
Acceptability	Interconnectivity	3	[30–32]
	Interoperability	3	[33–35]
	Semantic consistency	2	[36, 37]
	Convergent integration	10	[13, 38–46]
Cost-effectiveness	Interconnectivity	2	[31, 47]
	Interoperability	4	[48–51]
	Convergent integration	3	[43, 52, 53]
Data quality	Interconnectivity	2	[54, 55]
	Interoperability	8	[35, 56–62]
	Semantic consistency	8	[36, 63–69]
	Convergent integration	6	[40, 52, 70–73]
Flexibility	Interconnectivity	2	[74, 75]
	Interoperability	7	[33, 50, 57, 60, 76–78]
	Semantic consistency	2	[36–66]
	Convergent integration	4	[44, 79–81]
Positive predictive value	Interconnectivity	1	[82]
	Interoperability	6	[49, 50, 83–86]
	Semantic consistency	3	[87–89]
	Convergent integration	2	[90, 91]
Representativeness	Interconnectivity	1	[92]
	Interoperability	2	[58, 93]
	Semantic consistency	1	[64]
Sensitivity	Interconnectivity	10	[30, 55, 82, 92, 94–99]
	Interoperability	16	[10, 34, 50, 51, 55, 78, 83–86, 100–105]
	Semantic consistency	10	[37, 67, 68, 87, 89, 106–110]
	Convergent integration	8	[39, 40, 72, 80, 90, 111–113]
Simplicity	Interconnectivity	3	[31, 54, 114]
	Interoperability	2	[78, 115]
	Semantic consistency	2	[36, 106]
	Convergent integration	2	[44, 91]
Stability	Interconnectivity	1	[32]
	Interoperability	2	[115, 116]
	Convergent integration	1	[117]
Timeliness	Interconnectivity	7	[54, 96, 97, 99, 114, 118, 119]
	Interoperability	10	[10, 34, 48, 77, 93, 103, 116, 120–122]
	Semantic consistency	9	[63, 64, 66, 69, 89, 123–126]
	Convergent integration	15	[23, 40, 43, 70, 72, 73, 91, 113, 117, 127–130]

^a^**Data source:** PubMed, HINARI, Web of Science, Science Direct and advanced Google search engines

**Search strategy: PUBMED database**

1. Surveillance

2. Monitor

3. Monitoring

4. Monitored

5. 1 OR 2 OR 3 OR 4

6. Integrate

7. Integration

8. Integrated

9. Integrating

10. 6 OR 7 OR 8 OR 9

11. Animal health

12. Human health

13. Public health

14. One Health

15. 11 OR 12 OR 13 OR 14

16. System

17. Systems

18. 16 OR 17

19. 5 AND 10 AND 15

**Fig. 2:**
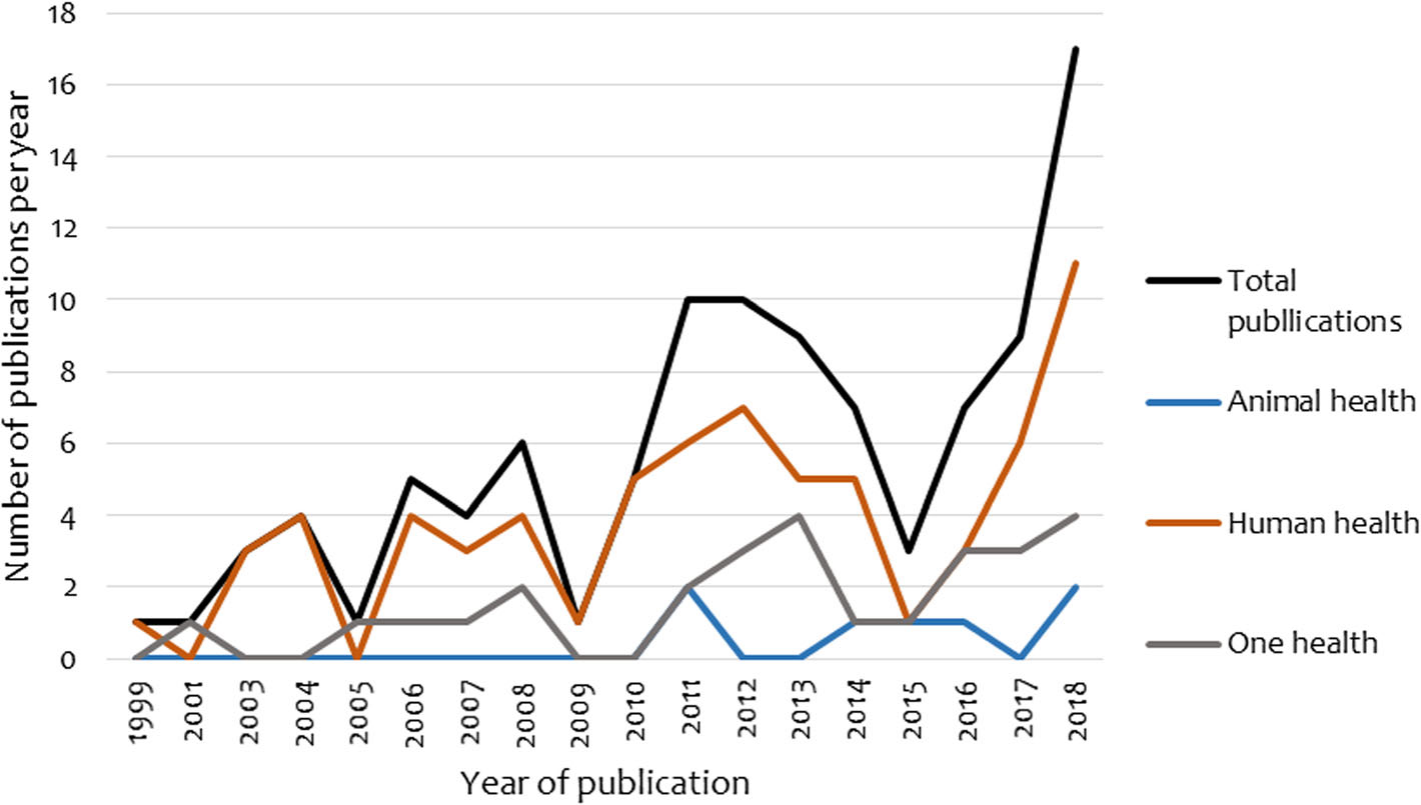
Distribution of the reviewed articles by year of publication

Of the 102 articles analyzed, 66.7% were specific to human health surveillance systems, followed by One Health (26.5%) and animal health (6.9%). Figure 2 shows the change in the annual number of publications between 1999 and 2018. The number of publications between 2011 and 2018 accounted for 62% of the total publications. Publications in human and One Health surveillance systems showed similar patterns from 2006 to 2018, Publications on animal health surveillance system integration were first spotted in 2011 and fluctuated thereafter with no clear trend.

### Health surveillance systems integration mechanisms

**Fig. 3:**
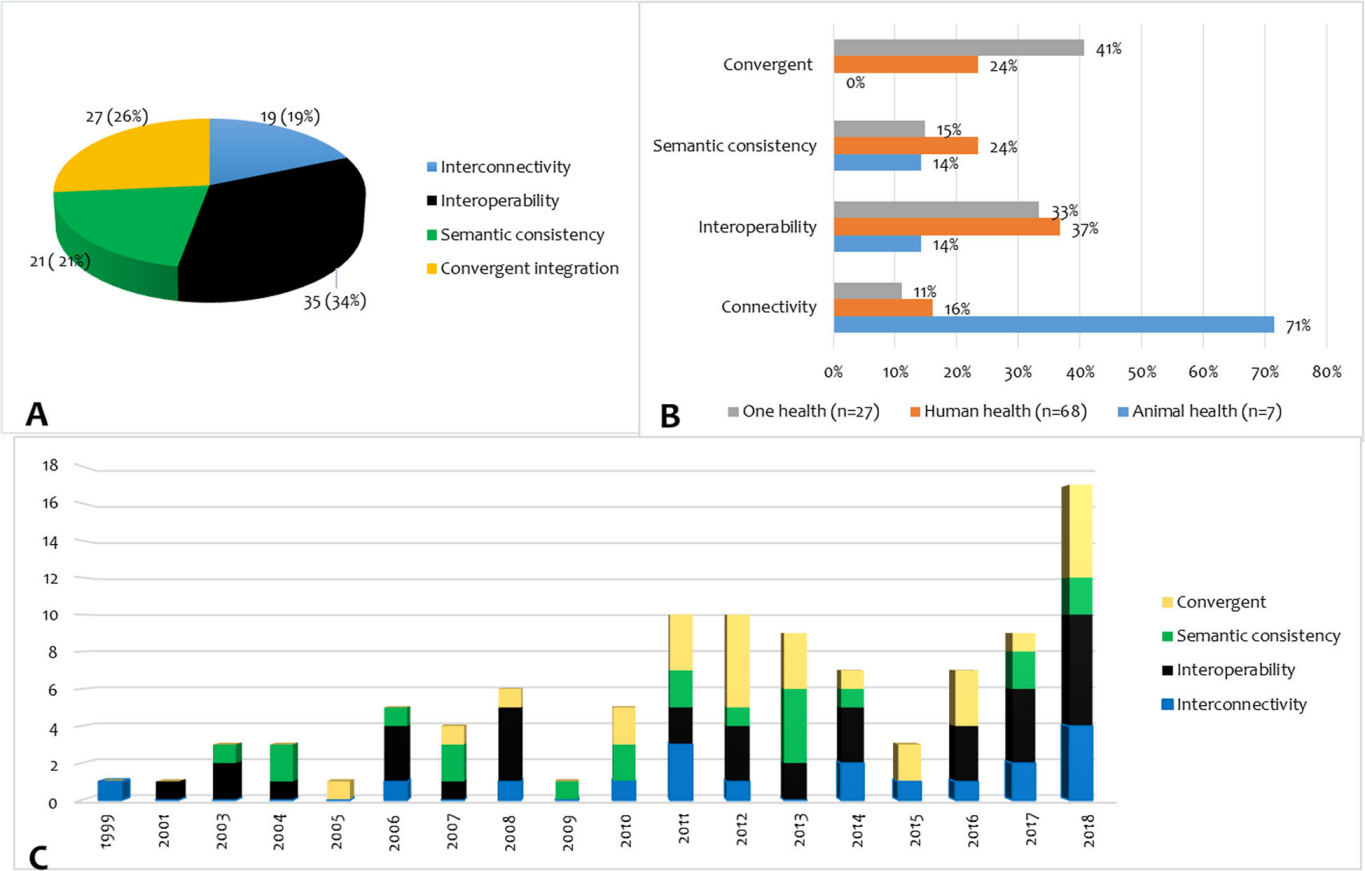
Surveillance system integration mechanisms. A = proportion overview of all mechanisms; B = distribution by sectors; and C = the trend of publications

The integration mechanism found were interconnectivity [19], interoperability [33], semantic consistency [21] and convergent integration [27] (Fig. 3a). Animal health surveillance systems used interconnectivity (71%) more than any other mechanism. One Health surveillance systems adopted mainly convergent integration (41%) and interoperability (33%) integration mechanisms. Human health surveillance system integrations were found to use more interoperability (37%), semantic consistency (24%) and convergent integrations (24%) (Fig. 3b). There was a higher number of publications on interoperability mechanisms for many years while convergent integration was first spotted in 2007 and the number of publications increased from 2010 though not consistently (Fig. 3c).

### Regional distribution of integrated surveillance systems

North America (33.3%; 34/102) and Europe (24.5%; 25/102) had a higher number of publications on the surveillance systems integration than other regions (Table 3). Australia (1.9%; 5/102) and South America (1.9%; 5/102) reported the lowest number of studies. Generally, there were more studies on human health surveillance across all regions than on animal or One Health surveillance. Despite the fact that North America had a large number of publications, no study was found on animal health surveillance. Of the seven reported studies on animal health surveillance systems, four (57.2%) were based in Europe. One Health surveillance was mostly reported in North America (9/27) and Europe (5/27).

**Table 3:** Distribution of surveillance system integration mechanisms by Regions (n = 102 articles)

		Region^a^								
Integration mechanism	Sector	Africa	Asia	Australia	Europe	North America	South America	International^b^	Unknown	Total
Interconnectivity (*n* = 19)	Animal health	0	1	0	3	0	0	1	0	5
	Human health	1	0	0	3	3	0	3	1	11
	One Health	0	0	0	2	1	0	0	0	3
	**Total**	**1**	**1**	**0**	**8**	**4**	**0**	**4**	**1**	**19**
Interoperability (*n* = 35)	Animal health	0	1	0	0	0	0	0	0	1
	Human health	2	1	2	7	10	2	0	0	24
	One Health	1	1	1	1	3	2	0	0	9
	**Total**	**3**	**3**	**3**	**8**	**13**	**4**	**0**	**0**	**35**
Semantic consistency (*n* = 21)	Animal health	0	0	0	1	0	0	0	0	1
	Human health	1	2	0	5	8	0	0	0	16
	One Health	0	0	0	1	3	0	0	0	4
	**Total**	**1**	**2**	**0**	**7**	**11**	**0**	**0**	**0**	**21**
Convergent (*n* = 27)	Animal health	0	0	0	0	0	0	0	0	0
	Human health	6	4	0	1	4	1	0	0	16
	One health	2	1	2	1	2	0	3	0	11
	**Total**	**8**	**5**	**2**	**2**	**6**	**1**	**3**	**0**	**27**
	**Total per region**	**13**	**11**	**5**	**25**	**34**	**5**	**7**	**1**	**102**

^a^The Region was determined by the country in which the system was located

^b^ International cover studies which were done in more than one region

Interoperability integration was mostly found in North America (14/34), Europe (8/25) and Asia (5/13). The highest number of studies in interconnectivity (8/19) and convergent (8/27) system integrations were found in Europe and Africa, respectively while semantic consistency was mostly practiced in North America (11/21) and Europe (7/21). Australia and South America had the lowest number of publications in all four integration mechanisms with no single study on interconnectivity or semantic consistency.

### Integration mechanisms on strengthening surveillance systems attributes

**Fig. 4:**
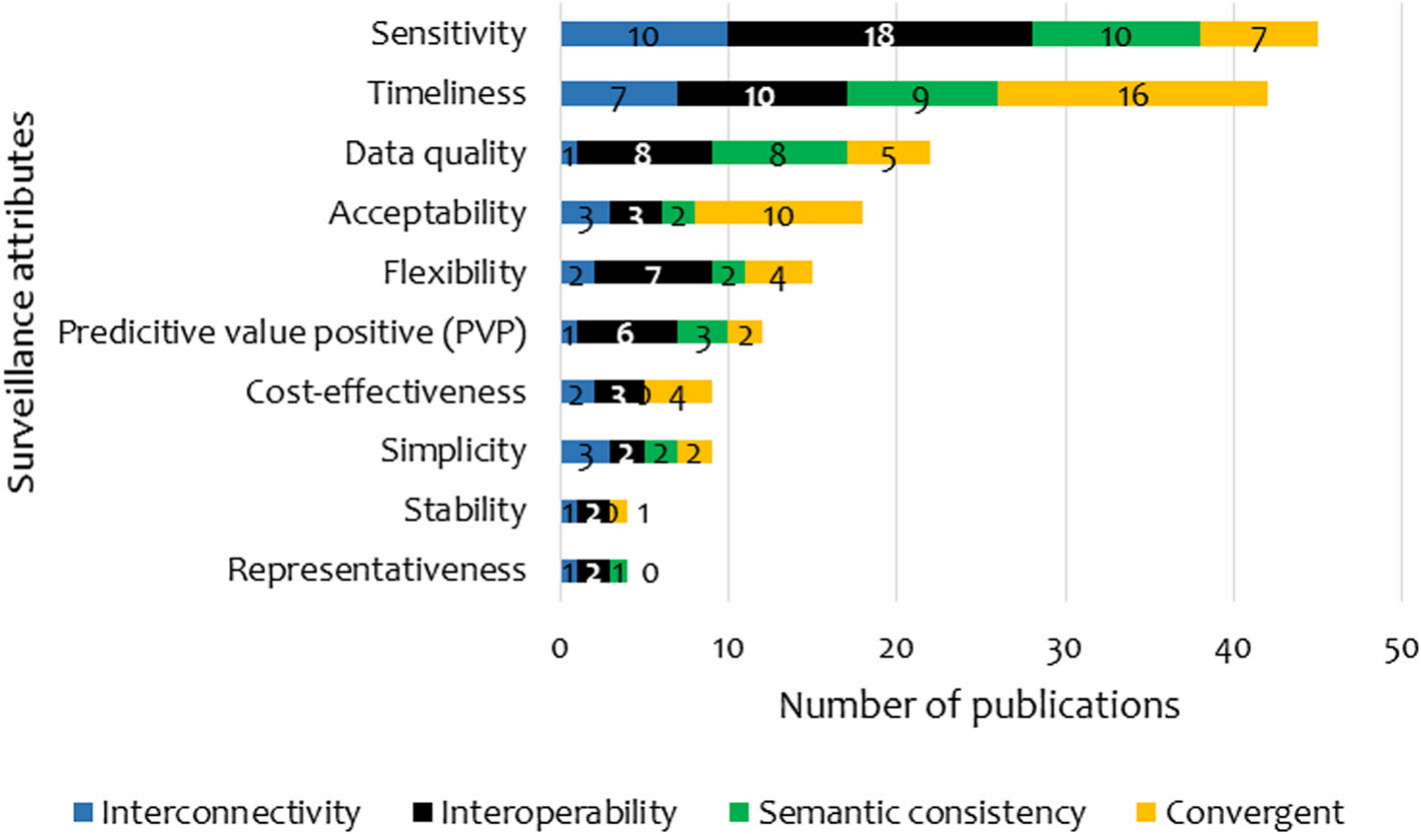
Distribution of surveillance system integration mechanisms by attributes

The systems integration attempted to improve at least one of the surveillance attributes. Most of the integrations focused on sensitivity (44.1%; 45/102), timeliness (41.2%; 42/102), data quality (23.5%; 24/102) and acceptability (17.6%; 18/102). Very few studies focused on improving stability (3.9%; 4/102), representativeness (3.9%; 4/102), cost-effectiveness (8.8%; 9/102), and simplicity (8.8%; 9/102) of the surveillance systems (Fig. 4). Convergent integration and interoperability were mentioned in relation to most surveillance attributes with a higher frequency for the timeliness, sensitivity, and acceptability for the former and timeliness, sensitivity and data quality of the latter. Semantic integration focused on sensitivity (10/21), timeliness (9/21) and data quality (8/21). There were fewer articles on the interconnectivity as one of the integration mechanisms and they mainly looked into sensitivity (10/19) and timeliness (7/19).

Of the 45 publications that focused on sensitivity, 16 quantified the performance of the attribute. Similar patterns were found for timeliness (8/42) and data quality (4/24). Overall, the sensitivity of the integrated systems reported in these studies ranged from 63.9 to 100% (median = 79.6%, *n* = 16) and data quality improved by 73 to 95.4% (media*n* = 87%, *n* = 4). The systems also managed to improve timeliness where the recorded changes were reported to be ranging between 10 to 91% (median 67.3%, n = 8).

### Value of health surveillance systems integration mechanisms in relation to disease control strategies

**Fig. 5:**
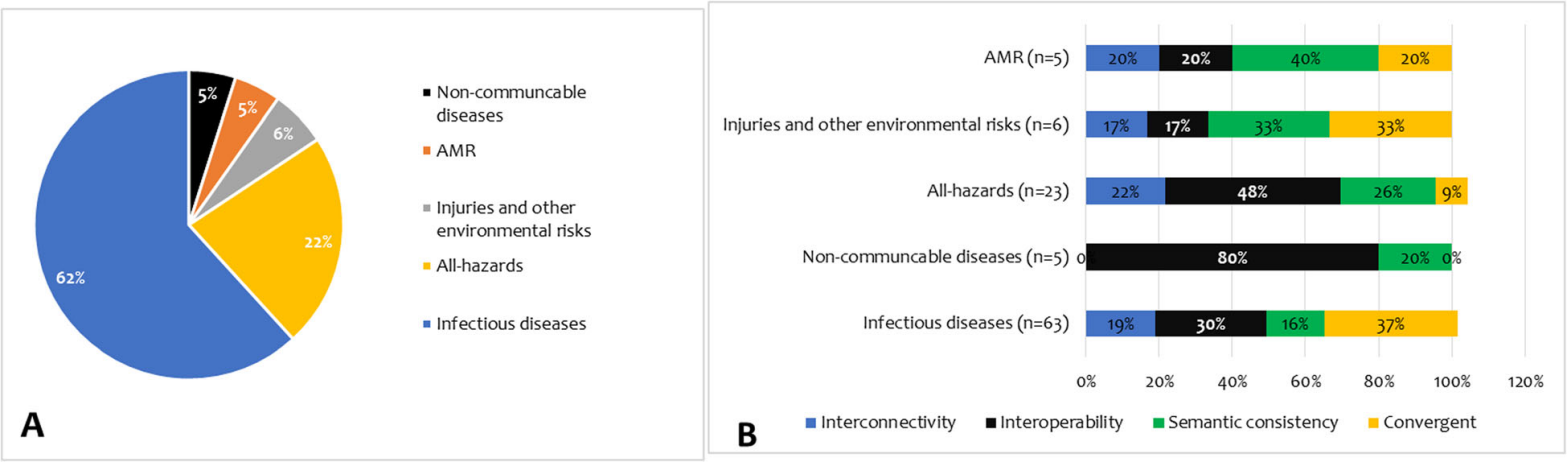
Distribution of surveillance system integration mechanisms by target hazards. A = distribution by hazards; B = distribution by integration mechanisms against hazards

Of the 102 articles, 62% focused on infectious diseases followed by all-hazards (22%). Surveillance on injuries and other environmental risks, antimicrobial resistance (AMR) and non-communicable diseases accounted for 16% (Fig. 5a). Interoperability and semantic consistency were the most adopted integration mechanisms. Nevertheless, there were variations across the target hazards (Fig. 5b). Convergent integration was reported more in infectious disease surveillance systems. Semantic consistency (40%) and interoperability (80%) were mostly found in AMR and non-communicable disease surveillance systems, respectively. Interconnectivity was fairly distributed in most of the target hazards.

### Challenges in surveillance system integration

A number of challenges have been identified (Table 4), some are common across all the integration mechanisms, whereas others are specific to a particular domain. The majority of the challenges are related to data management, compliance with standard operating procedures and financial resources.

**Table 4:** Challenges described for the four integration mechanisms (*n* = 29 studies)

Integration mechanism	Challenge	References
Interconnectivity	Heterogeneity of data sources may affect the results of the system.	[78]
	Limited knowledge of terms of reference, surveillance procedures, and case definitions.	[50]
	False positives.	[70, 95, 115]
Interoperability	Linkage and management of heterogeneous data.	[79, 80]
	Slow adoption of technologies.	[52]
	Limited resources.	[55, 111]
	The installation of systems can be complex and expensive.	[80]
Semantic consistency	Heterogeneity of data sources may affect the results of the system.	[121]
	Low compliance with standard operational procedures.	[63, 75]
	Incomplete integration.	[34, 53]
Convergent	Poor data management systems.	[66, 108]
	Limited laboratory capacity.	[66, 109, 125]
	Different data and reporting policies among participating institutions.	[108]
	Quantity and complex nature of data.	[76]
	Organizational and structural barriers.	[39, 123]
	Incomplete integration.	[68, 108]
	Limited awareness of the standard case definition of a disease.	[36]

## Discussion

This review identified 102 articles on human and animal health surveillance systems integration mechanisms and how they have been used to strengthen surveillance attributes. However, since the review was limited to publications in English, it is likely to have missed additional literature presented in other languages. The findings indicate that there is a substantially higher number of publications on human health surveillance integration compared to animal or One Health. The majority of the reviewed articles focused on infectious diseases. Integration in health surveillance was found to gain momentum during the current decade interoperability and convergent integration were the most frequently reported mechanisms of integration in health surveillance. Studies addressed one or more of the surveillance attributes but there was no study that reported on integration mechanisms in comparison to a large number of surveillance attributes. Similar results have also been reported elsewhere [29]. While the majority of the integration focused on improving sensitivity, timeliness and data quality, very few attempted to provide quantitative analysis on the performance of those attributes which may not suffice to make any conclusion on their impacts.

The finding that there are very few studies in animal health surveillance systems compared to human health or One Health concurs with findings reported by other authors [127, 131]. This can be linked to the fact that International community regards One Health as a more effective option for strengthening human, animal and environment health due to cost saving, ease coordination and efficient resource mobilization [132–134]. Hence, the reasons for more interventions being geared towards One Health surveillance rather than sectoral systems. However, while thinking of One Health surveillance, there should be parallel initiatives to strengthen sectoral surveillance systems, especially animal health surveillance as emphasized by another author [131]. Effectively integrated animal health surveillance systems are the cornerstones in addressing global security threats such as zoonotic diseases [135], antimicrobial and food safety [136]. If well integrated, animals can be used as surveillance tools for human and environmental health hazards [137].

System integration is relatively a new concept in health surveillance systems, especially in animal health. The spectrum of integration regards interconnectivity as the lowest and simplest integration mechanism while convergent is more complex yet the highest level of integration [15]. In this review, it was found that more integrations were towards interoperability and convergent integration mechanisms. This is likely to be attributed to the increased calls for more collaboration across sectors in addressing emerging and re-emerging zoonotic diseases [132, 133] which may also mean the integration of structures and harmonization of various operational procedures. On the other hand, interoperability is regarded as more convenient and safer for systems. This is because it does not require much merging of the system but rather synchronization where heterogeneous systems can be made networkable over a single physical network with the possibility of varying the degree of interoperation [15]. Integration of health systems has widely being used in healthcare service systems with diverse experience and outcomes [127, 138]. Majority of the integration initiative in health surveillance leverage on the existing healthcare information systems such as electronic medical records, birth and death registers, and laboratory information systems. Regardless of the mechanisms used, the integrated surveillance systems have shown a promising path towards addressing global health security threats. That is evident through the integration objectives [70, 111] adoption rate and level of efforts used especially on technological innovations and stakeholders’ involvement and some of the reported benefits such as improvement in sensitivity, data quality, and timeliness. Nevertheless, it is clear that there is no one-fits-all integration because most of them try to address one or a few attributes of the surveillance system.

There has been a significant increase in the number of publications on surveillance integration in recent years. One of the reasons may be because most of the systems were established only in the last decade [139]. This is likely to be linked to the rapid technological advancement and its active role in facilitating data capture, reporting, and analysis even in the resource-limited areas [8, 140, 141]. For instance, between 2000 and 2005, internet access improved more than 4-fold in low-and-middle-income countries, and more than a quarter of the population in these countries uses mobile phones [100]. The use of internet-based surveillance is both logistically and economically appealing [25]. Meanwhile, there is a paradigm shift in health surveillance where systems leverage on the rise of artificial intelligence to automate the process, allow the collection of data from a wider variety of sources and allow the dissemination of data to a wider audience [142, 143]. In the current era, social, technical and technological components are the key ingredients of any successful integration which clearly specify functions and performance of the designed system. High adoption of interoperability and semantic consistency mechanisms in Europe and North America can be associated with technological advancement and relatively stronger information systems [135]. Interconnectivity and convergent integrations were common in Europe and Africa. Technological disparities can affect the collaboration among the countries and penetration of sophisticated systems. While pacing towards meeting global targets on health security it is worth acknowledging the technology disparities between low-and-middle-income and high-income countries and the need for intensive investment in that area.

When integration mechanisms were assessed against surveillance systems attributes, sensitivity, timeliness, and data quality were found to be the central focus making about two-thirds of all reported attributes. Similar observations have been reported by Drewe et al. [29]. On the other hand, very few studies evaluated the effects of the integration in those attributes. However, strengthening of surveillance systems attributes should be well scrutinized and any modification should consider the system holistically because it may adversely affect other attributes of higher priority such as an increase in the cost of the system [142]. Nevertheless, it is worth mentioning that very few publications provided details on the evaluation of surveillance attributes, which limited the ability to reach strong conclusions on the efficiency of integration in strengthening the surveillance systems. It is critical that the evaluation of integrated systems should be comprehensive and consider relevance, efficiency, effectiveness, impact, and sustainability.

In the human health sector, IHR 2005 requires developing and maintaining core capacities in detecting and responding to an emerging threat in a timely manner [144]. The animal sector is guided by Terrestrial Animal Health Code which requires member states to carry out monitoring, surveillance, and reporting of animal disease outbreaks especially those listed as notifiable diseases to the World Organization of Animal Health [3]. A large portion of studies being focused on infectious diseases and all-hazards is an added value in line with international regulations. In the human health sector, IHR 2005 requires developing and maintaining core capacities in detecting and responding to an emerging threat in a timely manner. The percentage change is slightly higher than what was found in the electronic surveillance system [145]. This implies that the time lag between the onsets of the disease to its detection is shorter in the integrated system than in the conventional system. It is also evident that the integration of a surveillance system with technology improves the sensitivity of the system [139, 140].

Despite the evident role played by integrated surveillance systems in improving early detection and response, there are some critical issues to consider for them be functional and effective. The efficiency of the integrated surveillance systems is the function of data management systems, organizational structures, adequate resources (human, technology, infrastructure, and finance) diagnostic tools, clear standard operating procedures [38, 111, 136, 141] and political will. Data management systems should be accompanied by constant technological innovation in order to make sure the system accommodates as many data sources as possible [146, 147]. Organizational structures are of paramount importance in strengthening intra- and inter-institutional collaboration and communication regarding surveillance [135]. The structure should be able to accommodate both vertical and horizontal flows of information and be flexible enough to absorb challenges that may arise from the increased interdependence of the system components. Non- or low-compliance to standard operating procedures (SOP) and terms of reference is still a challenge. That is partly associated with diverse scenarios encountered during implementation [63], limited knowledge on the usage and lack of guidelines and relevant reference documents [29, 90]. Therefore, when considering integration of the system, standard operating procedures and terms should be made available and go hand in hand with capacity building and training to the users.

## Conclusion

This review showed that of the four integration mechanisms, interoperability and semantic consistency are the most common ones. It is also evident that systems integration in health surveillance is a relatively new concept that has been gaining the momentum in recent years. While few formal evaluations are available, integration mechanisms seem to have the potential to improve surveillance performance; more quantitative studies need to be conducted to confirm this. Technology advancement holds a large share in the future of surveillance systems integration. For successful implementation and operation of surveillance systems integration, technology innovation and strengthening of data management systems are needed to link and manage large amounts of heterogeneous data. Evaluation of the integrated systems should be comprehensive and consider relevance, efficiency, effectiveness, impact, and sustainability.

## Supplementary information

**Additional file 1.**

**Additional file 2.**

## Data Availability

Contact the authors for any additional information.

## Abbreviations

AMR: Antimicrobial Resistance
IHR: International Health Regulation
PRISMA-P: Preferred Reporting Items for Systematic Reviews and Meta-Analysis Protocols
USA: United States of America
WHO: World Health Organization
OIE: Office International des Epizooties
